# Blockchain-Based Distributed Information Hiding Framework for Data Privacy Preserving in Medical Supply Chain Systems

**DOI:** 10.3390/s22041371

**Published:** 2022-02-10

**Authors:** Abir EL Azzaoui, Haotian Chen, So Hyeon Kim, Yi Pan, Jong Hyuk Park

**Affiliations:** 1 Department of Computer Science and Engineering, Seoul National University of Science and Technology, Seoul 01811, Korea; abir.el@seoultech.ac.kr (A.E.A.); chahot@seoultech.ac.kr (H.C.); hyeonos248@seoultech.ac.kr (S.H.K.); 2 Department of Computer Science, Georgia State University, Atlanta, GA 30302, USA; yi.pan@siat.ac.cn

**Keywords:** blockchain, Information Hiding Techniques, medical supply chain, privacy, security

## Abstract

Medical supply chain communication networks engender critical information and data. Notably in the COVID era, inner personal and private information is being shared between healthcare providers regarding the medical supply chain. In recent years, multiple cyber-attacks have targeted medical supply chain communication networks due to their lack of security measures. In the era where cyber-attacks are cheaper and easier due to the computational power and various algorithms available for malicious uses, security, and data privacy requires intensive and higher measures. On the other hand, Information Hiding Techniques (IHT) compromise various advanced methods to hide sensitive information from being disclosed to malicious nodes. Moreover, with the support of Blockchain, IHT can bring higher security and the required privacy levels. In this paper, we propose the implementation of Blockchain and smart contract with the information hiding technique to enhance the security and privacy of data communication in critical systems, such as smart healthcare supply chain communication networks. Results show the feasibility of the framework using Hyperledger smart contract along with the desired security level.

## 1. Introduction

Critical Internet of Things addresses the time-critical and data-critical systems that demand higher security levels and deal with highly confidential information. Smart healthcare supply chain communication networks engender various private medical data including personal information about the patient and their medical history, which make it a part of critical IoT systems. Moreover, the healthcare providers communicate sensitive information regarding their supplies between each other, notably during the COVID era, where hospitals were obliged to share their reserve of medical supplies and follow the supply chain information of the vaccine. Following the Electronic Health Record (EHR) system, healthcare providers are encouraged to share most of their patient information with each other under the act of Health Information Exchange (HIE) [1] to help improve the provided services in hospitals. Patient Health Information (PHI) and supply chain information are at a high risk of data breaches and theft. Cybercriminals can easily target the communication channels or the database that contains millions of patients’ personalized data and history. In 2014, the Community Health System (CHS) was the target of the Heartbleed attack, which cost the data and information exposure of more than 4.5 million patients [2]. Healthcare data are mostly stored in local databases or cloud-based databases, and in both cases, they require higher protection against any possible attack. Still, hospitals and healthcare providers seem to spend far fewer investments on technology support and security options. Not to mention the fast development of mobile healthcare systems that allow the patient to access their data directly via a tablet or smartphone, the market size of mobile healthcare in China reached a peak of 2.9 billion yuan in 2015 [3], which indicates the high dependency on mobile healthcare and its deployment to access critical data, such as EHR by the patients. Moreover, patients themselves can help in leaking their data by sending it via unprotected channels and without encryption.

Critical IoT systems including healthcare providers have to improve their security measurement all while maintaining the desired data communication speed, to reach this goal, multiple states-of-arts have studied various techniques for enhancing the security and privacy of data including Blockchain, intensive encryption methods, and so on. One of the most promising methods is the Information Hiding Technique (IHT). Information hiding techniques deploy the method of inserting data into another one to confuse eavesdroppers and cyber-attackers [4]. Most of information hiding techniques utilizations are in copyright enforcement, securing data over unsecured communication channels, and data manipulation detection [5].

In this paper, we deployed an improved Steganography method that includes hiding the desired information in another form of data, such as text or image. We implement Blockchain, as well to create a cluster of pre-authenticated and honest healthcare providers to securely communicate with each other while limiting the risk of eavesdroppers. A smart contract is used as well to automatically generate a one-time secret key that will be known by only the participants in the Blockchain-based cluster to enhance the privacy of communications. In our proposed framework, the desired message, or data to be communicated shall be encrypted with other auxiliary text messages, that are useless both to the sender and receiver, in form of blocks of messages.

The main key contribution to our paper can be summarized as follows:

- We deploy an improved version of steganography techniques to encrypt the desired communicated messages and information into another auxiliary message to enhance the privacy and security of the communicated medical supply chain information against any possible cyber-attack.
- We deploy Blockchain technology to create a secure and private cluster of pre-authenticated healthcare providers. Only nodes included in the cluster may participate in the communication, view the received messages, and decrypt the desired block while ignoring the auxiliary blocks.
- Finally, the smart contract is implemented in our proposed framework to automatically generate one-time secret keys and distribute them between the concerned parties securely. The secret key changes with every new communication initialization, eliminating the risk of cyber-attacker having the key to decrypt the information, thus enhancing the security and privacy of critical systems, such as smart healthcare.

The rest of the paper is organized as follows: Section 2 presents a detailed background study on the deployed technologies including Blockchain, IIoT system, and Information Hiding Techniques. In Section 3, we propose our novel Blockchain-based IHT framework with a methodology to explain our concept. While Section 4 supports our proposed framework with extensive system analysis and results. Finally, we conclude this work in Section 5.

## 2. Related Works

The recent advancement of networks and fast speed of computational algorithms makes it easier for malicious nodes to attack the IIoT system with Ransomware and other cyber-attacks to paralyze the IIoT networks and smart factoring. In this section, we discuss first the key technologies in this paper including Blockchain technology, IIoT systems, and Information Hiding Techniques.

### 2.1. Background

To provide secure data communication for critical IoT, several technologies and solutions can be used, we discuss some of the possible solutions in the related works section. However, in this paper, we propose the fusion of Blockchain with IoT security systems for private and secure communication between multiple layers over the classical channel. To this end, in this section, we will define the deployed technologies

#### 2.1.1. Blockchain

Blockchain is a decentralized technology that uses public-key encryption to provide identity authentication and records every transaction for every identity in a P2P network, which is irrevocable once the transaction is completed. Each node in the network records all the transaction information of the distributed network in which it is located and is packaged into blocks in chronological order. Each new block is time-stamped and proceeds in a strict timeline sequence, with blocks connected in an irreversible chain. Each block in the blockchain contains the hash value of the previous block, and a cyber attacker who wants to tamper with the information in the blockchain needs to tamper with all the new blocks after the block. The blockchain adopts a one-way hashing algorithm which makes any attempts to hack and tamper with the data information within the blockchain easily traceable [6]. The nodes in a blockchain system are in a decentralized, distributed P2P network, and broadcast messages arrive at the nodes at different times. An attacker can use this delay to deliver fake messages to neighboring nodes in faster unicast communication, and blockchain systems provide a consensus mechanism to avoid this problem. We hope that the consensus mechanism can provide sufficient credibility to all nodes in the distributed network. Each node in the network can publish information to other nodes. When other nodes in the network receive the broadcast issued by a node authenticated by the consensus mechanism, we consider this message to be trusted and unforged information and package this information into a new block and add it to the current chain of blockchain. Consensus nodes in decentralized systems are self-interested, and maximizing their benefits is the fundamental goal of their participation in data verification or bookkeeping. The blockchain system rewards nodes that complete the consensus mechanism to ensure that each node in the network is willing to participate in the data verification under the consensus mechanism [7]. Public Blockchain is a blockchain that any node in the network can access and participate in a consensus mechanism. In a public blockchain, as long as a node completes the algorithmic requirements of the consensus mechanism, its recorded data can be added to the Blockchain, and corresponding economic rewards can be obtained. The public blockchain-based network can be seen as completely decentralized, and thus, have more evil nodes in the network. Public blockchain ensures the reliability and security of data by cryptography economics and generally uses cryptography verification combined with consensus mechanisms, such as proof of work or proof of ownership to ensure trusted access [8].

#### 2.1.2. Critical IoT Systems

There are some challenges in the Internet of Things, such as centralized management structures that cannot prove their innocence; with the geometric growth of IoT devices, the centralized structure of the future cannot afford such massive data connections. Moreover, much of the Internet of Things is a self-organizing network within carriers and enterprises. The cost of establishing credit is high when it involves collaboration across multiple operators and peers [9]. Blockchain multicenter, weak centralized characteristics will reduce the high operational costs of centralized architecture, information encryption, secure communication characteristics will help to protect the privacy and identity rights management and multilateral consensus helps to identify illegal nodes and promptly to prevent malicious nodes access, relying on the chain structure will help to build a card can be traced back to a shred of electronic evidence exists, The distributed architecture and peer-to-peer characteristics help to break the shackles of multiple information islands existing in the Internet of Things and promote the horizontal flow of information and multi-party collaboration [10].

The IoT System is a globally distributed network system and refers to a network technology in which devices are connected and communicate with each other through the Internet. Devices with IoT technology sometimes collect data through sensors and share it with other devices to create valuable data and results. Representative IoT technologies that can be met are smart home, health monitoring, and indoor localization. Industrial IoT is a subcategory of IoT and refers to IoT technology used in industrial fields. The scope of application of the technology is large, such as factories and power plants, and it is used for smart logistics, remote maintenance, and intelligent factories. As it is used in industrial sites, the overall scale is large, so there is a sensor scale, and its mobility is weak.

IIoT uses the basic technology of IoT. In IIoT, wired/wireless devices are connected and used on a large scale, and this includes sensors and mobile communication or Wi-Fi networks. Each device communicates with the user through the sensor and then transmits this communication information to the cloud through the network. A place where data is stored close to the user is called an edge, and because data is shared over the network through this, it is called edge network and edge computing. The simplified structure of cloud/edge computing is a “picture”. Compared to cloud computing, which required data from the network to the cloud, data is stored and provided at the edge in the middle, enabling quicker responses to users.

#### 2.1.3. Information Hiding Techniques

Advanced security methods and solutions, such as cryptography and data concealment are deployed to safeguard critical information from cyber threats. The key usage of cryptography is to turn the plain text into a ciphertext that is impossible to understand or decipher without the secret key [11,12].

On the other hand, Information hiding techniques are used to hide critical information and data in another form of data, such as text or image, making sure the documents are not tempered with using watermarking. Information hiding techniques and cryptography are both used to protect the information. However, they are not quite similar. There are two main categories of information hiding, notably steganography and watermarking. Steganography is used to hide the data in a different form of other data, such as media or text. While watermarking is defined as the process of concealing data in digital files so that it can withstand changes and revisions to protect the intellectual property of digital media [13].

### 2.2. Existed Solutions for IoT and Medical Supply Chain Security

The advent of tiny, low-cost sensors and high-bandwidth wireless networks means that even the smallest devices can now be connected, with a certain level of digital intelligence. They are monitored and tracked, their status data is shared, and they communicate with other devices. All of this data can then be collected and analyzed to improve the efficiency of the business process. IIoT should not be confused with the consumer Internet of Things, but the core concept of the consumer Internet of Things is the same as IIoT, using sensors and automation to improve efficiency. If the application of the Internet of Things in the industrial industry is abstracted, we can summarize it into four levels: data collection and display, basic data analysis and management, in-depth data analysis and application, and industrial control. Table 1 summarizes the key points of the quantitative study.

Sengupta et al. [14] sorted out the security threats and corresponding solutions based on the four levels of the Internet of Things, as well as various network attacks and blockchain-based solutions based on different targets in the Industrial Internet of Things. Security objectives in the Industrial Internet of Things can be broadly divided into infrastructure security, authentication and trust protocols, authentication modes, secure data management systems, and traditional attack prevention strategies.

The Industrial Internet of Things places more emphasis on the collection and sharing of limited and specific information between companies. In traditional supply chains, new orders are sent to suppliers by fax or Courier mail. Combined with the IIoT, the supply chain can be deployed as a hands-free network of resource interactions where every sensor in the supply chain is automated, providing automation for a range of processes in the supply chain involving product production and distribution.

Wen et al. [15] proposed to ensure that data does not leak when sensors collect data, IIoT devices will be upgraded to the blockchain by storing monitoring and recording of IIoT devices in real-time in the network through smart contracts.

Iqbal et al. [16] propose how IIoT and blockchain can work together in industrial processes to solve security problems in real-time. It also designs the security requirements of IIoT and blockchain and describes how IIOT can be integrated into blockchain for intelligent industrial applications. The DEMATEL method was used to calculate the economic challenges faced by the industrial Internet of Things in the industry and address the cost of infrastructure design and deployment through blockchain factors.

Zhao et al. [17] divide blockchain nodes into full nodes (FN) and light nodes (LN). FN can download and inspect all blocks and transactions and can act as a mining node to create blocks for the blockchain. Due to resource limitations, LN can store and process some data on the blockchain. In the Industrial Internet of Things, LN participates in new transactions that propagate between nodes and can be added to a block in the blockchain. The framework very much combines the scalability requirements of IIoT and the operational model of mass data processing.

Yu et al. [18] show that to meet the requirements of secure storage, access control, information update and deletion of intelligent factory data, as well as tracking and cancellation of malicious users, the author designed the industrial Internet data sharing mechanism based on the blockchain. The secure storage and sharing of smart factory data in the cloud are enhanced by using blockchain to support identity authentication and traceability. In this mechanism, the key volume is small, the operation efficiency is high and it can resist collusion key attacks.

Wu et al. [19] propose the use of blockchain combined with edge computing to solve the scalability and data security problems of the existing industrial Internet of Things. Edge computing provides new node deployment options for blockchain. Blockchain is deployed on edge computing nodes, which facilitates data docking and controllable transmission paths, alleviates bandwidth pressure, improves real-time transmission, and integrates the development capabilities of operators to improve the overall operational efficiency of IIoT. In addition, blockchain can promote the coordination and synchronization between different edge nodes, help establish the integrity guarantee of edge computing systems and anti-counterfeit storage support resources, promote the open sharing of terminals, data, and capabilities, so as to provide trusted services for vertical industries.

Liu et al. [20] propose a performance optimization framework for blockchain-based IIoT systems to optimize scalability/throughput while considering system decentralization, security, and latency. In order to deal with the dynamic and complex characteristics of the industrial Internet of Things system, DRL is used for deep reinforcement learning to effectively process massive data. The framework can increase operational efficiency while ensuring IIoT security. Each block producer and consensus algorithm is evaluated according to the proposed quantitative measurement system of blockchain system performance, and the block size and block interval are adjusted by DRL technology to maximize the on-chain transaction throughput of the blockchain system.

Guan et al. [21] divide the energy trading model into two layers, namely BC-ETS, a blockchain-based energy trading solution that protects privacy and balances power supply and demand at the same time. In addition, in order to adapt to the relatively weak computing capacity of the underlying IoT devices in the energy Internet, a fair-proof mechanism based on honesty is designed to greatly improve the availability of the system. BC-ETS not only meets security requirements but also provides high performance for energy trading solutions.

Ghadge et al. [24] defined the traditional supply chain risk and summarized how organizations manage network risk in the supply chain. From the network risk type; Network risk dissemination; Network risk penetration point; Cyber security challenges and mitigation measures using connection-based clustering to identify and validate guidance and inform the analysis. Data mining techniques were used to build models for comprehensive, reproducible, and transparent reviews. Complementary clustering analysis based on data mining provides transparency and rigor for the implementation of the first SLR of supply chain network risk.

Helo and Hao [25] have developed a prototype of a blockchain-based Logistics Monitoring System (BLMS). In this BLMS, logical data is collected and shared through a blockchain solution. The functionality of the system enables customers and logistics operators with all other partners to track and track their data across the ecosystem and obtain their own data information from the system. The high availability and security of blockchain applications in the supply chain system are emphasized.

Bhaskar et al. [26] showed the current COVID-19 pandemic has exposed the supply chains and response capabilities of agencies. The global medical supply chain system requires large-scale, open, and innovative approaches that cushion the system during emergencies and long-term needs and ensure the continuity of essential health care supplies and the resilience of health care systems. Technologies, such as blockchain, can be used as drivers to further improve operational efficiency.

Musamih et al. [27] studied the challenges of drug traceability in the pharmaceutical supply chain and emphasized its importance, especially for anti-counterfeiting drugs. They developed and evaluated a solution for tracking and tracking drugs in a decentralized manner in a blockchain-based platform pharmaceutical supply chain. Their proposed solution leverages the cryptographic underpinning of blockchain technology to enable tamper-proof logging of events in the supply chain and smart contracts in the Ethereum blockchain to enable automatic logging of events accessible to all participating stakeholders. The primary goal is the integrity, availability, and non-repudiation of transaction data, which is critical in complex multi-party environments, such as pharmaceutical supply chains.

Carmody et al. [28] address vulnerabilities being exploited in individual software components of healthcare technology. The risk of including third-party software components in healthcare technology can be managed in part by utilizing the Software Bill of Materials (SBOM). SBOM provides a transparent mechanism to ensure the security of the software product supply chain by identifying and fixing vulnerabilities more quickly, thus achieving the goal of reducing the feasibility of attacks. SBOM has the potential to benefit all supply chain stakeholders of medical technology without significantly increasing software production costs.

Garcia-Villarreal et al. [29] surveyed the existing supply chain management literature and classified the work of Critical Success Factors (CSF) and the sectoral focus of medical technology. Divided into (1) sales and operations planning, (2) product development processes, (3) quality and compliance, (4) procurement strategy, (5) customer relationship management, and (6) production systems. The results of this study indicate that the combination of these CSF resulted in better performance by Original Equipment Manufacturers in the medical technology sector. Highlighting the outstanding factors of supply chain management success that have not been fully recognized in previous studies, in particular: quality and compliance, product development processes, and production systems. Supply chain improvement is not about implementing isolated projects, but about carefully selecting appropriate and targeted measures to allocate resources more efficiently and efficiently.

Pandey et al. [30] pointed out that the impact of network physical system (CPS) vulnerabilities on network security mainly focused on the vulnerabilities of CPS. Based on information flows up and down the supply chain, the authors attempt to identify cybersecurity risks in the global supply chain. These network security risks are further classified from a strategic perspective. The conceptual model of network security risk and network attack of the global supply chain is proposed. These risks can be further verified and scaled up through empirical studies. From a management perspective, the framework can act as a decision-making process that simultaneously considers different cybersecurity risks at all stages of a globalized supply chain.

## 3. Proposed Blockchain-Based IHT for Critical IoT Security Framework

The main contribution of this paper includes the absolute security of data communicated between the IoT device layer and the cloud layer. In this case scenario, we considered medical IoT devices as a critical environment. Medical IoT holds sensitive data including patient personal information, such as name, disease history, and so on. Moreover, the data hold hospitals and laboratory research information and data, such as drug discovery data. These data need to be computed and processed in the cloud layer. Thus, the communication between the medical IoT layer and cloud layer needs to be highly secure.

### 3.1. Proposed System Overview

**Figure 1:**
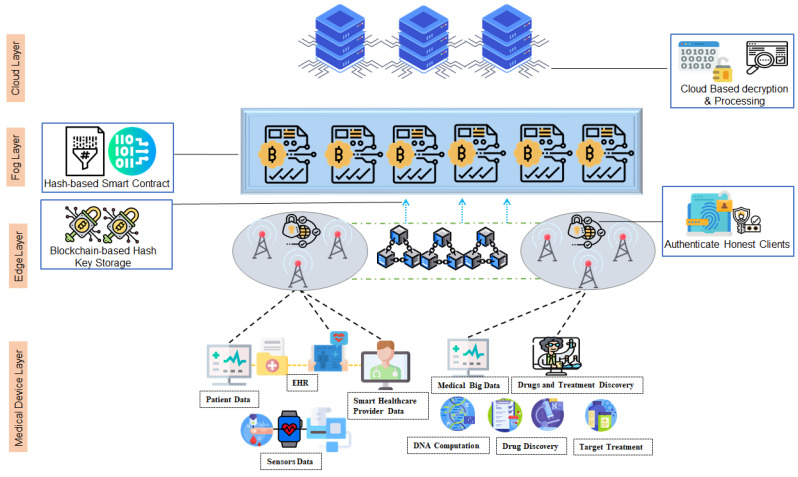
Proposed Blockchain-based IHT Architecture.

As shown in Figure 1, he presented Blockchain-based IHT framework can be divided into four main layers: (1) Healthcare IoT device layer, (2) Edge layer, (3) Fog layer, and finally (4) Cloud layer. In this section, we will discuss in detail the role of each layer.

Medical IoT device layer: The medical IoT device layer engenders various heterogeneous information that urges high-level security. For this case study, the type of data we opt to secure is the hospitals and laboratory research data, such as drug discovery data and patients’ information. The hospitals are generals and medical laboratories specifically process a large amount of data in cloud servers in order to find the results they look for, for instance, drug discovery laboratories may use patients’ personal medical history and information in order to proceed with their experiments. This information is usually communicated from the laboratory to the cloud server over classical channels and using basic encryption methods. At the edge layer, we implement a private Blockchain ledger for authentication and secure cluster selection purposes. The Base station plays the role of the Blockchain manager, and it has to verify the identity, authenticate users, and register honest ones into the ledger for a faster future authentication mechanism. The device user and its respective cloud server have to be registered into the Blockchain as well and they agree upon a unique hash key, the hash key will be stored into Blockchain for further encryption. We will discuss this phase in detail in the upcoming section. The fog layer engenders smart contracts between the device user and the cloud server. The smart contract deploys the previously discussed hash key in order to encrypt the information. Moreover, a secret message and auxiliary bits are encrypted as well to confuse the attacker in case of a cyber-attack, thus, the attacker cannot differentiate between the real message and the auxiliary encoded message. Several distributed cloud servers reside at the cloud layer. Their role is to process the received information, after decrypting it using the previously agreed on hash key. The hash key change with each communication round, making it a one-time hash, thus enhancing the security and privacy of the data.

### 3.2. Methodology of the Proposed System

In our proposed framework, we took under consideration the scenario where a new node (hospital, laboratory) requests to join the Blockchain-based system and share and receive information with other nodes. Under this scenario, we depicted our case study in three main phases.

The first one is the selection of a secure cluster where only pre-authenticated and validated healthcare providers nodes can join the secure network to share the information and communicate with each other, this phase is realized using Blockchain private ledger and PBFT algorithm that uses the previously authenticated and honest nodes to verify and validate the new ones. The second phase is the key hashing and registration phase, where all the pre-authenticated nodes from the previous phase have to agree on a secret key to use during the communication, hash it, and broadcast it securely between each other. Note that the key changes every time the communication session is initialized, this provides a higher security level similar to the one-time pass algorithm. The final phase is the smart contract phase where the pre-agreed key hashes are stored in a smart contract and executed automatically with every new communication session, the smart contract is also responsible for modifying the secret key with every message automatically, making it easier and highly secure for the execution of the key hash and communication encryption over classical channels. To explain the aforementioned steps, we consider a new node, a hospital, for example, requesting to join the Blockchain-based secure system. First, the hospital initialed PBFT algorithm, if the network reached consortium state, the hospital will be added to the Blockchain-based secure network, otherwise, the initialization is aborted. After the first phase is completed, the user device in the newly joined node has to create a secure key to encrypt its communicated data with other nodes. The generated initial key has to be computed and the output is a secure hash key that will be registered in the smart contract. In order to enhance the security level of data, the communicated messages include the original message, auxiliary message, and a secret message to confuse the eavesdropper in case of a cyber-attack. The message is divided into multiple segments and encrypted using the one-time hash key registered in the smart contract, then sent throughout the Blockchain network.

**Figure 2:**
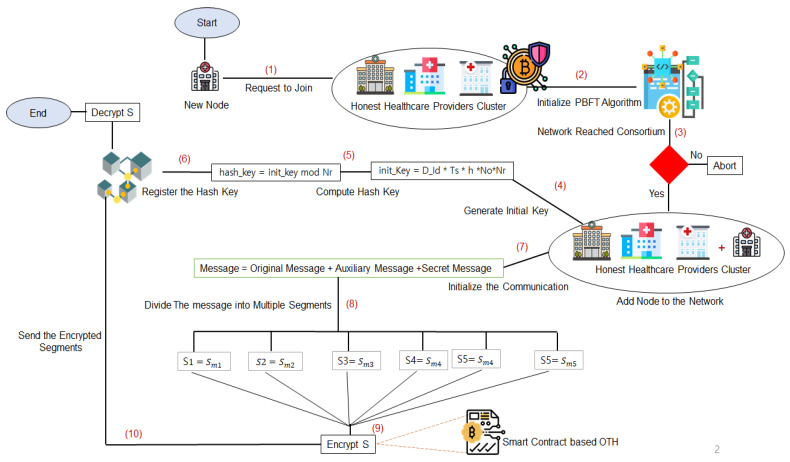
Methodology of the Proposed Framework.

The details of these phases can be depicted In the following sub-sections. Figure 2 depicts the methodology of the proposed framework with all the main phases including cluster registration, hash key computation, and smart contract-based One-Time Hash (OTH). The details of the methodology are explained in algorithms 1, 2, and 3 with their respective discussion.

#### 3.2.1. Cluster Pre-Selection Phase

The verified and authenticated healthcare providers are allowed to send a request and join the Blockchain-based communication system. Blockchain as a distributed, secure, and cryptographically guaranteed neither non-falsified nor modified distributed ledger technology [31,32,33,34,35,36,37] can be used as a means of verification and authentication of nodes. For this study, we deploy Blockchain to verify the healthcare providers and create a secure communication network channel; The deployed consortium algorithm is an enhanced version of Byzantine Fault Tolerance (BFT). A private Blockchain is deployed where the nodes that check and authenticate the requests are considered to be the head government healthcare center. Algorithms 1 and 2 depicted the required steps.
**Algorithm 1. Cluster pre-selection (message Broadcast).**1: **Input:** Healthcare provider identification and request
2: **Output:** Final consensus decision (Authenticated or rejected)
**3: Process:** 4:  $HC_{k}$.Send(<$CL_{ID}$, Request, Ts>, VC);
5:  VC. Check (<$CL_{ID}$, Hi, Request, Ts>, $HC_{0}$);
6: $HC_{0}$.Prepare(<w, Hi, Di>, Request);
7: $HC_{0}\;$.Communicate (<w, Hi, Di>, $HC_{N}$);
8: **for** i = 1 to n {
9:     $HC_{i}$.Get(<w, Hi, Di>);
10:    $HC_{i}$.Check(<w, Hi, Di>, f, n);
11:    $HC_{i}$.Prepare(<w, Hi, Di>);
 12:    $HC_{i}$. Communicate (<w, Hi, Di>, $HC_{\left|n-i\right|}$);
 13:    $HC_{i}$.count(<f: fault>, count C); }

The honest client selection can be explained as follows; when the healthcare provider wants to benefit from the secure communication, it has to send a request to the Validation Center (VC) first. In this algorithm, the VC can be a government healthcare authority that manages other clients; the CA is assigned the role of the root in this algorithm.
**Algorithm 2. Cluster pre-selection (message count and execute).**1:  **while** count: C > (f + 1) then 2:   $HC_{i}$.Stamp(<w, in: Di, out: Di, Ts, Stamp: k>); 3:   $HC_{i}$.Commit(<w, Hi, Di, Ts>, Result: k, $CL_{0}$); 4:   $HC_{i}.$Get(<w, Hi, Di, Ts>, Request: x, count C); 5:  **while** count: C>(2f+1) then6:    $HC_{i}.$Compute(<w, Hi, Di>, Request: x, $HC_{k}$= 0.Mark = x); 7: ** for** k = 1 to n8:    $HC_{i}$. Check(VC .Stamp = x);9:    if $HC_{k}$. Stamp == $HC_{0}$.Mark then 10:     add$\;HC_{k}$ToBlockhain (BC, $HC_{id}$, 0, $HC_{k}$); 11:  **else**
12:    skip ;**End**

The VC checks the identification of the healthcare providers; if it is authentic, VC takes the role of the head $HC_{0}$ and start communicating the request. It is imperative to mention that the sensors among a node are considered honest and are not prone to cyber-attacks, thus communicating only genuine data, as future scope for this work, a case scenario where the sensors are vulnerable to various attacks and the communicated messages are deceptive shall be considered and studied. The nodes that receive the request form $HC_{0}$ continue forwarding it and counting the number of messages in their memory.

After the number of messages exceeds f + 1 round, the commit status is communicated, and the marked results are sent to $HC_{0}$. The function Stamp is used for final verification. The communication continues until it reaches 2f + 1 rounds. Using the Stamp function, the results of $HC_{0}$ and $HC_{k}$ can be compared. If results are consistent, the healthcare provider is added to the Blockchain network, if not, the request is omitted.

Following Algorithms 1 and 2, we ensure that only honest clients can be a part of the system, thus providing the desired protection for smart healthcare communication. Therefore, only honest and verified clients can utilize this system.

#### 3.2.2. Hash Key Registration Phase

Before starting the communication phase, the device user and its respective cloud server have to agree on a shared hash key to be used at the beginning of the communication in order to encrypt the data, for this end, Algorithm 3 depicts the phases of the hash key registration.
**Algorithm 3: Hash key registration.**1:  Input: IoT devices, Cloud server
2:  Output: Secure shared hash key
3:  Process:
4:  **Start:**    //Initialization
5:  M_IoT. Send (<D_Id, Ts, Msg, h, No>, CS);   // with D_Id is the IoT device identification,
   6: Ts is the timestamp, No is the nonce, h is the height of the message
   7: CS is the cloud server
8:  CS. Receive(<D_Id, Ts, Msg, h, No>);
9:  CS. Check (<D_Id, Ts, Msg, h, No>);
10:   **if** Device. Existe == true
{
11:     CS. Generate (<init_Key <= (D_Id * Ts * h *No*Nr))       //with Nr is the number of communication round
12:     CS. Send (<init_Key>, M_IoT)
13:     M_IoT.Compute (<hash_key>, init_key mod Nr)
14:     CS.Generate(<New Block>)
15:   **if** CS.hash_key == M_IoT.hash_key
{
16:     CS.Add (hash_key, M_IoT>, New Block)
17:   **else**
18:     CS. Abort (Communication);
}
}
19:  **end;**

After registering the hash key into Blockchain, it will be used during the communication phase to encrypt the message. Each time the communication is initialized, the hash key change due to the change of *Nr* (number of rounds), thus, creating a new secure hash key shared only by the IoT device and its respective cloud layer.

Here, we deploy a smart contract to execute the hash encryption and encrypt the message. The data sent by the IoT device, or the healthcare provider can be represented as follows:

 Message=Original Message+Auxilary Message+Secret Message 

The original message engenders the data sent for computation and processing by the cloud server, while the auxiliary message is a string of bits message sent as noise to confuse the eavesdropper in case of a successful cyber-attack. The auxiliary string and secret message cannot be decrypted by the cloud server, additionally, they are encrypted using the device’s private key, thus, the server can ignore them. However, if an attacker managed to access the system and view the message, it will be confusing as the auxiliary message is meaningless. The smart contract will execute the encryption phase using the hash key agreed on in the previous phases.

## 4. Analysis and Discussion

To evaluate the first step (Blockchain-based Cluster Selection), we used Network Simulator-3 (ns-3), which relies on C++ to implement the smart city network models and Python for network topology. IBM Hyperledger was deployed to implement the Blockchain and smart contract. The simulation was performed on an intel core-i7 computer with 16 GB of RAM running under Ubuntu Linux.

### 4.1. Analysis

**Figure 3:**
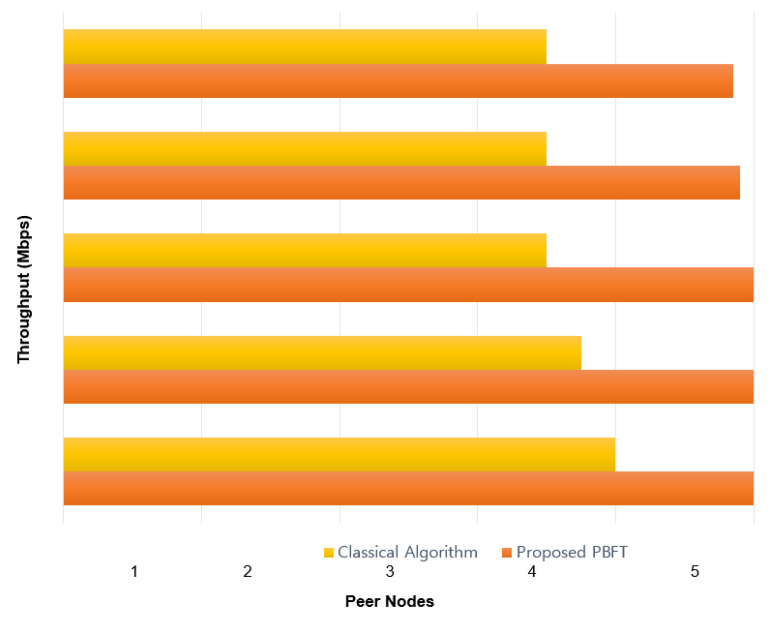
Throughput Comparison between Classical BFT and Proposed PBFT.

In order to prove the effectiveness of our proposed architecture based on Blockchain, we take into consideration three main network performance metrics; latency of fault peers, general latency of execution over the network, and network throughput using PBFT compared with the classical algorithm. The proposed Blockchain algorithm shows better results than the classical PBFT algorithm in terms of execution. As depicted in Figure 3, we ran the simulation on NS-3 with GO-Ethereum under the case scenario of four-fault peers, to generate private communication networks for data. The obtained results prove the feasibility of this architecture using Blockchain technology. The proposed solution records a low latency and high throughput compared with the classical BFT algorithm.

Moreover, the implementation of smart contracts over Ethereum shows a maximum latency of 60 s, which is not ideal for critical systems, such as smart healthcare communication. Smart healthcare communication requires fast and efficient data transmission between sender and receiver as the life of a patient can be in danger if more delay happens. To this end, we implemented the smart contract that generates the new hash key automatically over the IBM Hyperledger fabric.

**Figure 4:**
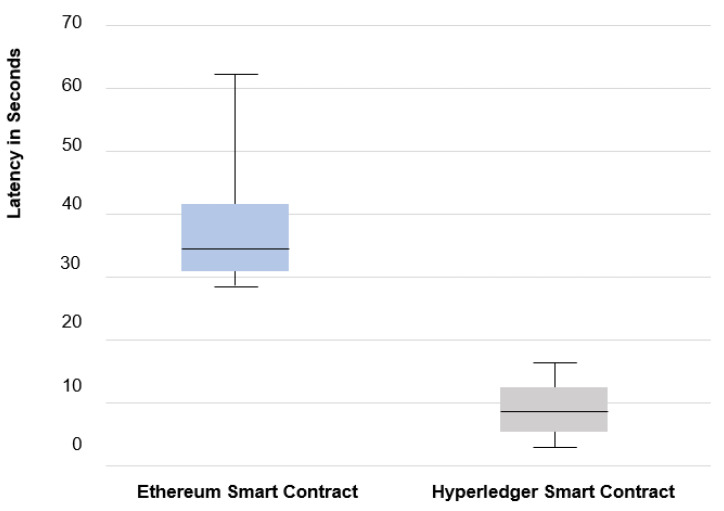
Latency Comparison Between Ethereum based Smart Contract and Hyperledger based Smart Contract.

Hyperledger Fabric is an open-source project based on the Linux Foundation to develop an enterprise-level application. A smart contract allows an automatic generation of OTH. The hash changes after each round of communication based on the number of rounds, thus ensuring a higher security level and limiting the chances of an eavesdropper or cyber-attacker finding the respective hash key. In this case, the scenario generated hash key is only known by the participants in the same Blockchain-based cluster. The original message is the only segment encrypted using the smart contract generated hash, while the auxiliary segments are encrypted using the sender chosen method to confuse the attacker. Once the segments are received, the receiver will apply the smart contract OTH on them, and the segment that is successfully decrypted using the OTH decryption key is the one containing the original message, while the others are ignored. Figure 4 depicts the results of execution time using both Ethereum and Hyperledger fabric based on our simulations and shows that the Hyperledger fabric-based smart contract is faster and more feasible for critical systems, such as smart healthcare.

Moreover, the implementation of the proposed framework guarantees the achievement of five fundamental aspects to the medical supply chain system including consistency where the medical supply chain system remains consistent and updated in real-time due to the fast and secure data communication between deferent components and participants. Security is another major key requirement of the proposed framework, communicated data should be protected and secured from any possible cyber-attacks. The proposed solution ensures the security of information using Blockchain and a one-time hash-based smart contract. The third aspect is availability, the system has to be available for data communication and information retrieval all the time. This is possible by using a decentralized Blockchain network where the information is prone to a single point of failure. On the other hand, integrity is another parameter to be considered as well. The integrity of the system is secured using a one-time hash-based smart contract, where the encryption key automatically changes with each round of communication. Finally, the proposed framework maintains the transparency of data and information in the medical supply chain as it can be tracked and registered into the Blockchain network and shared between all the participant nodes.

### 4.2. Discussion and Open Research Challenges

The proposed framework deploys an information hiding technique that uses an auxiliary message to hide the original message in a critical environment, such as smart healthcare systems. The message is divided into multiple segments and encrypted using a smart contract-generated one-time hash. Moreover, to enhance the security and privacy of the system, we use a Blockchain-based private cluster. The role of the cluster is to select honest healthcare providers, verify, and authenticate them using an improved version of the BFT algorithm. The healthcare providers in one cluster are able to communicate patient information and data between each other, and the cluster head to control the communication is considered to be a government center. The healthcare providers then generate an initial key and register it into the Blockchain. Using the initial key, a smart contract can compute a new hash key with each communication phase and execute it automatically. The sender creates a list of segments that includes the original message and addition of auxiliary messages, only the original message segment is encrypted using the pre-agreed on OTH, while the other segments are encrypted using the sender chosen method. The message is sent via Blockchain to the receiver, which by its turn applies the OTH decryption key into the received segments. The segment with the original message shall be decrypted accordingly, while the auxiliary messages shall be ignored. This method can be applied not only during the communication phase but also while storing the data in a local or cloud-based database. Hiding the original data into useless texts or images proves to be highly secure as the segments are encrypted differently, which will take a longer time for the attacker to decipher without the right key.

Other existing states-of-art can be merged with this proposed framework to enhance the security and privacy of the medical supply chain, to mention some, Niu et al. [38] discussed location privacy-preserving methods against long-time observation attacks. Their proposed framework named Eclipse creates a balance between location privacy and the usability and availability of the system. Although it is not mentioned in their paper, the proposed solution can effectively be implemented in medical supply chain services as well to enhance the security and privacy levels. On the other hand, unlike most related works, Jin et al. [39] proposed a novel mobile crowdsensing system framework that automatically selects workers that provide reliable data. This method can be used as well during the phase of honest nodes selection in our proposed framework. Finally, Jia et al. [40] implemented an online privacy-preserving on-chain certificate status named PROCESS using Blockchain. This method can be merged with our framework to verify the certificate of the validation center, thus enhancing the security of our system.

The limitation of this work resides in the encryption and decryption phases; both phases are not discussed in detail as we proposed a general overview of the idea in the paper. As future work, we intend to depict in detail the encryption and decryption phases and test the proposed framework over multiple and various systems, such as smart factories and smart homes. Critical systems require higher and tighter security measures, and with this solution, we can improve the security and privacy of smart systems and cloud-based databases.

## 5. Conclusions

Information Hiding Techniques (IHT) engenders various advanced methods to hide sensitive information from being disclosed to malicious nodes following multiple techniques. IHT is useful to protect the privacy and authenticity of communicated messages, data files, and even electronic contracts between companies. Moreover, with the support of Blockchain as a decentralized distributed ledger that promises non falsifying and non-tampering with the blocks, Blockchain-based IHT can bring higher security and privacy levels for critical networks requirements. In this paper, we propose the implementation of Blockchain and smart contract to the information hiding technique to enhance the security and privacy of data communication in critical systems, such as smart healthcare. Blockchain is deployed to create a secure private cluster of honest healthcare providers to communicate, while the smart contract is used to automatically generate an OTH for encryption purposes. As for the information hiding technique, we use a segment that includes the original message hiding in auxiliary messages; only the original message is encrypted using the OTH, while the auxiliary message is encrypted using various encryption techniques chosen by the sender and can be ignored by the receiver. Our proposal proves lower execution time compared with classical techniques and promises higher security measurement. As future work, the encryption and decryption techniques of OTH shall be depicted in detail with real-case scenarios of other critical systems.

## Figures and Tables

**Table 1:** Related Works Comparison Table.

Research Work	Year	Technologies	Key Contribution	Limitation
Sengupta et al. [14]	2020	Blockchain Smart Contract Classification	Summarize security issues of blockchain-based IoT and IIoT	No case study was proposed
Wen et al. [15]	2019	BSCS ABE	Solve supply chain resource interaction security issues	Lack of comparative results
Iqbal et al. [16]	2020	Blockchain DEMATEL	A detailed study to evaluate IIoT performance and reduce the cost of infrastructure deployment	Propose only a study of existing solutions
Zhao et al. [17]	2019	Blockchain Smart Contract Identification technology	A comprehensive survey on Blockchain scalability and data capacity of IIoT	Propose only a study of existing solutions
Yu et al. [18]	2021	Blockchain	Enhanced secure storage and sharing of smart factory data in the cloud	The time cost of the encryption phase is slightly higher than other works
Wu et al. [19]	2020	Blockchain Edge Computing	Solve the scalability and data security problems of the Industrial Internet of Things	Lack of comparative results
Liu et al. [20]	2019	Blockchain DRL	Optimize IIoT scalability/throughput.	Lack of comparative results
Guan et al. [21]	2020	BC-ETS	Balance power supply and demand while protecting the privacy	System communication overhead is slightly higher than related work
Panchal et al. [22]	2018	IoT, IIoT, Security, CPS	A comprehensive survey on the security threats on IIoT and various attacks on the layered IIoT architectures	Propose only a study of recent states-of-art
Mabkhot et al. [23]	2018	Big Data, Cloud, Manufacturing, IoT, CPS, Smart Factory	Analyze and identify perspectives of the smart factory.	Propose only a study of recent states-of-art
Ghadge et al. [24]	2019	Data mining	Defines the traditional supply chain risk and summarizes how the organization manages the network risk in the supply chain.	No case study was proposed
Helo et al. [25]	2019	Blockchain, BLMS	Study of a blockchain-based logistics monitoring system (BLMS).	Lack of comparative results
Bhaskar et al. [26]	2021	Blockchain	Discuss the core development and innovation needs of the medical supply chain	Lack of simulation results
Musamih et al. [27]	2020	Blockchain	Developed and evaluated a solution for decentralized tracking of drugs in a blockchain-based platform pharmaceutical supply chain	Latency and throughput of the proposed solution are not considered in the simulation
Carmody et al. [28]	2021	Software Bill of Materials	Addresses vulnerabilities exploited in individual software components of healthcare technology	Lack of security analysis
Garcia-Villarreal et al. [29]	2019	Big data	Discuss outstanding factors of supply chain management success that have not been fully recognized by previous studies	No case study was proposed
Pandey et al. [30]	2020	Big data, deep learning	A comprehensive study on network security risk of global supply chain	No solution was proposed
Our Work	2021	Blockchain, One-time hash-based smart contract	Privacy-preserving in the medical supply chain using Blockchain and smart contract	Only tested on medical supply chain-based scenario

## Data Availability

Not applicable.
